# *In vitro* and *in vivo* effects of fungicides on angular leaf spot pathogen (*Pseudocercospora griseola* f. *griseola*) and common bean

**DOI:** 10.7717/peerj.21477

**Published:** 2026-07-03

**Authors:** Sirel Canpolat

**Affiliations:** Plant Disease, Directorate of Plant Protection Central Research Institute, Ankara, Türkiye

**Keywords:** Common bean, Fungal disease, Germination, Seed treatment, Phytotoxicity

## Abstract

Angular leaf spot, caused by *Pseudocercospora griseola*, is a destructive fungal disease inducing yield losses up to 80% in common bean-growing areas of tropics and subtropics. Although various fungicides are used against the disease, their potential side effects on host plants are not well-documented. This study aimed to assess efficacy of six commercial fungicides (azoxystrobin, carbendazim, chlorothalonil, difenoconazole, mancozeb, and thiram) with different action mechanism and chemical groups against *P. griseola in vitro*, and to evaluate their effects on seed germination and seedling growth of common bean (cv. Gina) *in vivo*. Solid nutrient medium containing the fungicides were used for *in vitro* experiments, while seed germination tests described by International Seed Testing Association (ISTA) were used for *in vivo* experiments. As a result, all the fungicides significantly (*P* ≤ 0.01) inhibited conidium germination *in vitro* and their effectiveness were ranked from the highest to the lowest one as follows: difenoconazole, azoxystrobin, mancozeb, carbendazim, thiram, and chlorothalonil, respectively. However, *in vivo* results showed that the tested fungicides significantly reduced seed germination of common bean compared to controls, but carbendazim and chlorothalonil caused the highest reductions, 23.7% and 23.5%, respectively. Fungicide treatments also significantly increased abnormal germination rate. Furthermore, all the fungicides led to notable reductions, 24.4% and 68.4%, in emergence rates and plant height, respectively. These findings indicate that although all the tested fungicides can inhibit conidium germination of *P. griseola in vitro*, they also exhibit significant phytotoxic effects on common bean *in vivo* by negatively affecting seed germination, seedling emergence, and growth.

## Introduction

Common bean (*Phaseolus vulgaris* L.) is very important legume with protein, mineral and antioxidant content in human diet globally (Oblessuc et al., 2012; Karavidas et al., 2022). However, production of the crop is adversely affected by *Pseudocercospora griseola* causing yield losses up to 80% in common bean (Fontem, Nziengui-Nziengui & Bikoro, 2007; Ddamulira, 2019; Páramo et al., 2024). Losses from angular leaf spot was estimated as 384.2 tonnes per year in Sub-Saharan Africa as well (Kijana et al., 2017).

Angular leaf spot frequently occurs in tropics and subtropics where common bean is grown (Landeras et al., 2017; Nay et al., 2019). It is considered as one of the major fungal diseases of common bean in South Africa, Brazil, Argentina, Tanzania, Ethiopia, Democratic Republic of Congo, East and Central Africa (Boshoff et al., 1996; Viecelli et al., 2010; Chilagane et al., 2016; Mongi et al., 2018; Gudero & Terefe, 2018; Aytenfsu, Terefe & Ayana, 2019; Taboada et al., 2022; Ruhebuza et al., 2024). Apart from this, it was reported over 60 countries in the world (Fritsche-Neto et al., 2019). For example, severe infection rates ranging from 60% to 100% were reported in common bean fields of Spain (Landeras et al., 2017) and in greenhouse grown common bean in the Western Black Sea region of Türkiye (Canpolat & Maden, 2020a). In addition, incidence and severity of the disease may reach up to 100% in most African countries as well (Pamela, Mawejje & Ugen, 2014; Kijana et al., 2017).

*Pseudocercospora griseola*, the causal agent of angular leaf spot, is previously also known as *Isariopsis griseola* and *Phaeoisariopsis griseola* (Pacific Pests, Pathogens, Weeds and Pesticides, 2025; PlantwisePlus, 2025). Two major groups, Andean and Mesoamerican, in *P. griseola* were designated as *P. griseola* f. *griseola* and *P. griseola* f. *mesoamericana*, respectively (Saparrat et al., 2009). *P. griseola* penetrates leaf through stomata and grow intercellularly between mesophyll and palisade cells (Monda, Sanders & Hick, 2001) and symptoms appear as angular spots on leaf and reddish-brown to dark spots on pod, stem and petiole of common bean (Landeras et al., 2017). The fungus reduces net photosynthetic ratio, transpiration ratio and stomatal conductance in infected leaves (Bassanezi et al., 2002). Spread of *P. griseola* occurs by airborne conidia released from spots on the infected leaves, while long distance spread of the fungus occurs through seeds (PlantwisePlus, 2025); therefore, seeds have a crutial role in the transmission of the fungus (Icishahayo et al., 2007; Jadon, Thirumalaisamy & Kumar, 2020). In a study, of 400 common bean seeds analyzed, 53% were infected by *P. griseola*, indicating prevalence and pathogenicity of the fungus in the seeds (Canpolat & Maden, 2020b). *P. griseola* is considered as harmful organism transmitted by infected seeds within the European Quarantine Regulation for many years. For this reason, European and Mediterranean Plant Protection Organization (EPPO) prepared a standard with coded PM 2/73(1) regarding this fungus. This standard has declared that management programs should be designed by considering inoculum sources (seeds, plant debris, air currents and water movements during irrigation) of the fungus to prevent the spread of the disease to clean areas (Eppo PQR Database, 2005).

Considering the seedborne nature of *P. griseola* and the EPPO standard mentioned before, using seeds certified or free of *P. griseola* is the main cultural management practice and seed treatments with fungicides are essential to prevent its spread *via* seed (Ayesha et al., 2021; PlantwisePlus, 2025). Therefore, seed treatments with fungicides may provide protection against seed-borne fungi in all growth stages of plants, ensuring healthy development and high yield (Moumni, Brodal & Romanazzi, 2023). A wide range of fungicides (*e.g*., carbendazim, azoxystrobin, difenoconazole, propiconazole, tebuconazole, flutriafol, mancozeb, metalaxyl, mancozeb, chlorothalonil, tridemorph, dinocap, fentin hydroxide, thiophanate methyl) have been used to control angular leaf spot (*Phaeoisariopsis griseola*) of common bean in legume production (Rajappan & Yesuraja, 1999; Fontem, Nziengui-Nziengui & Bikoro, 2007; Pamela, Mawejje & Ugen, 2014; Hennipman et al., 2019; Chandrashekara et al., 2022; Wani et al., 2022; Gorshkov et al., 2023). However, the fungicides used also raises concerns around the world due to their adverse effects on the environment and human health (Moumni, Brodal & Romanazzi, 2023; Grecco et al., 2024). Moreover, fungicides may hamper certain physiological and biochemical pathways during seed germination and plant growth as well (Singh & Sahota, 2018). A better understanding of interactions between fungicides and legume is very important to improve new control practices for fungal diseases of legumes (Shahid et al., 2018; Gorshkov et al., 2020, 2023). Due to the increasing use of fungicide-treated seeds, studies investigating impacts of seed treatments on seed characteristics are also needed (Carvalho et al., 2020). In recent years, although side effects of insecticides and herbicides on plant growth and development have been well studied, very limited study is available in literature related to effects of fungicides on plants. Even there is no knowledge about effects of fungicides on seed germination and seedling growth of common bean. In this context, the objective of this study was to evaluate and compare the *in vitro* antifungal efficacy of six commercial fungicides (difenoconazole, azoxystrobin, mancozeb, carbendazim, thiram, and chlorothalonil) with different action mechanism and chemical groups and a *Trichoderma harzianum*-based biological agent against *P. griseola*, while simultaneously examining their *in vivo* effects on seed germination, seedling growth, and potential phytotoxicity in common bean, under controlled conditions.

## Materials and Methods

### *In vitro* experiments

#### *Fungicides, biological preparation and P. griseola* f. *griseola isolate*

**Figure 1:**
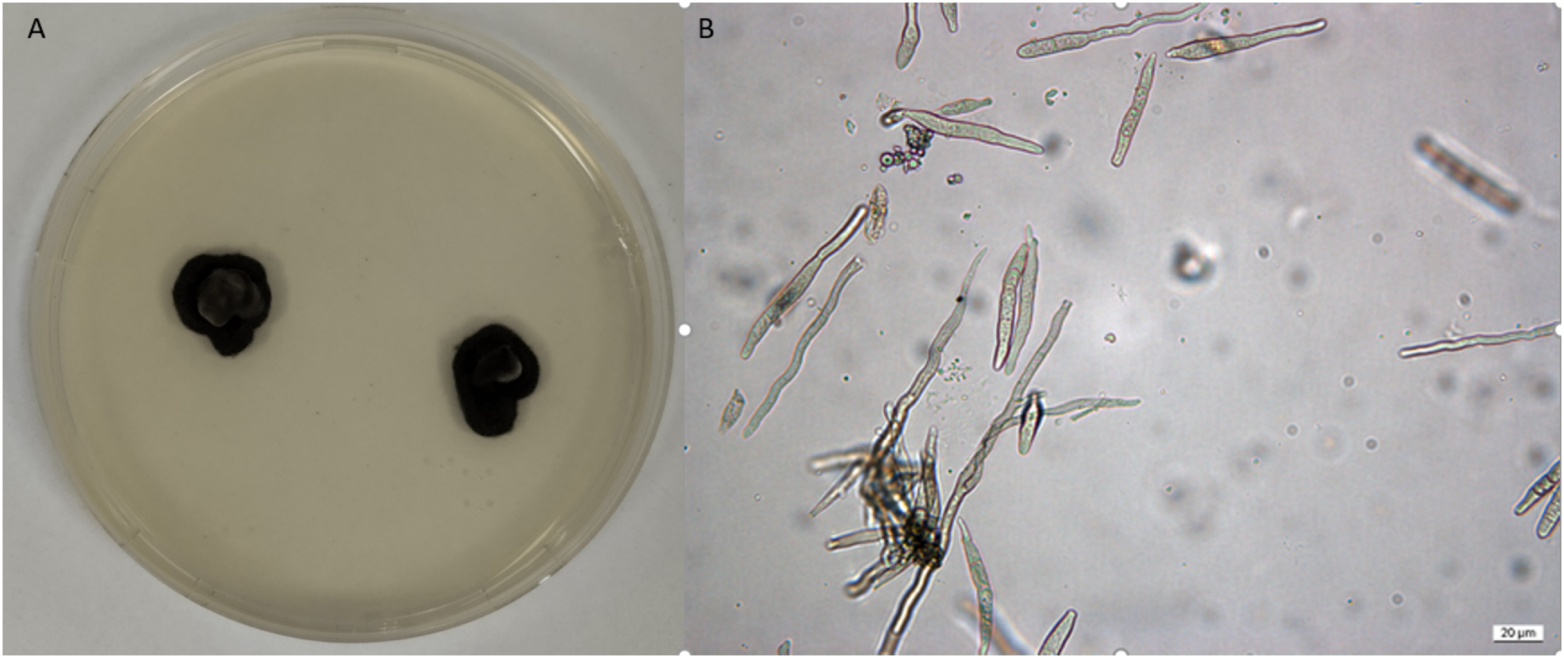
Colony development and conidia of *P. griseola f. griseola* SC120 isolate on potato dextrose agar (PDA).

Six commercial fungicides (azoxystrobin (Quadris 250 g/l, Syngenta, Basil, Switzerland), carbendazim (Sandazim 50 WP, Pileryum Tarım, Niğde, Türkiye), chlorothalonil (450 g/l, Koruma Klor, Derince, Türkiye), difenoconazole (Dividend, Syngenta, Basil, Switzerland), mancozeb (Fumazin M-45, Hektaş, Kocaeli, Türkiye), and thiram (Horn Forte, Agrotez Tarım, Istanbul, Türkiye)) and one biological preparation of *Trichoderma harzianum* Rifai KRL-AG2 (T-22 Planter Box, Hasel Tarim, Antalya, Türkiye) were used for *in vitro* experiments. SC120 isolate (Genbank accession no: MT445214) of *P. griseola* f. *griseola* with high pathogenicity (Canpolat & Maden, 2021) were used to determine efficacy of the fungicides against *P. griseola* f. *griseola* in the *in vitro* (Fig. 1).

#### Conidium germination tests

Conidium germination tests were performed based on solid nutrient medium containing the fungicides. To achieve this, initially, ten concentrations of 0.01, 0.03, 0.1, 0.3, 1, 3, 10, 30, 50 and 100 µg mL^−1^ of each fungicide were prepared using dilutions with distilled sterile water. Later, potato dextrose agar (PDA) was autoclaved at 121 °C for 20 min and cooled to 46 °C. Each adjusted concentration of each fungicide was put into the PDA medium and the same process was separately done for all the concentrations for each fungicide tested. Ten mL of PDA medium containing fungicides were poured per petri plate (9 cm) and left for solidification of the medium. In controls, fungicides were not put into PDA.

Fifteen mL of sterile distilled water was put on colony of the SC120 isolate of *P. griseola* f. *griseola* developing on PDA and then scraped with a spoon and filtered through a 2-layer sterile cheesecloth. Spore suspension of the isolate was adjusted to 1 × 10^6^ conidia/mL using a hemacytometer. Ten µL of spore suspension was dropped on the PDA containing the fungicides prepared before, while the same amounts were put on the PDA without fungicides for controls. These processes were separately performed for each fungicide application and experiments were conducted according to completely randomized design with three replicates. Petri dishes were kept in the dark at 24 °C for 14–18 h for conidial germination. To assess the germination, a total of 100 conidia per replication were examined by considering their germinations.

### *In vivo* experiments

#### Fungicides and common bean cultivar

The six commercial fungicides (azoxystrobin, carbendazim, chlorothalonil, difenoconazole mancozeb and thiram) and one biological preparation (*Trichoderma harzianum*) used in the *in vitro* experiments were also tested for their effects on seed germination and seedling growth of common bean (*Phaseolus vulgaris* L.) *in vivo* experiments (Table 1). Common bean cv. Gina, widely cultivated and highly adapted to Western Black Sea conditions of Türkiye, was used as host plant in the *in vivo*.

**Table 1:** Application/recommended rates, formulation forms of the fungicides and one biological preparation of *Trichoderma harzianum* used in the *in vivo* experiments.

Active substance and its ratio	Recommended rate	Formulation form	Application dose mL/g a.i./kg seed	
			1^st^ dose	2^nd^ dose
Azoxystrobin 250 g/L	250 mL/100 kg seeds	SC	0.1 mL	0.2 mL
Carbendazim %50	200 g/100 kg seeds	WP	0.08 g	0.16 g
Chlorothalonil 450 g/L	500 g/100 kg seeds	SC	0.4 g	0.8 g
Difenoconazole %2	100 g/100 kg seeds	DS	0.16 g	0.32 g
Mancozeb %80	200 g/100 kg seeds	WP	0.08 g	0.16 g
Thiram %80	200 g/100 kg seeds	WP	0.08 g	0.16 g
*Trichoderma harzianum*	7.5 g/100 kg seeds	WP	0.16 g	0.32 g

#### Fungicide applications

The seeds were treated with two lower doses of the fungicides than their recommended rates in Table 1, while in the controls seeds were only treated with water without fungicide application. To prepare the treatments, WP formulations were weighed and adjusted according to their required application rates. Later, adjusted amounts were added to the seeds in jars and mixed by shaking vigorously for 15 min. Liquid formulations were measured and diluted in 50 mL of water according to the required application doses. Afterwards, they were sprayed onto the seeds with sprayer and homogeneously mixed in jars. After these applications, the seeds were dried on filter blotter papers at 25 °C for 4 h and stored in glass jars.

#### Seed germination tests

Seed germination and emergence tests were conducted using plastic germination test boxes. In this respect, 100 fungicide treated seeds were placed on moist germination blotter papers in the plastic test boxes. In controls, 100 seeds treated only water without fungicide were placed on the blotter papers in the boxes. Experiments were carried out according to completely randomized design with four replications for each fungicide and its dose. The prepared test boxes were kept at 24 °C in germination cabinets. During the incubation of the seeds, the humidity in the test boxes was controlled and water was added to the dried ones, and the seeds were evaluated at the 5th and 9th days of the incubation. The evaluation of normal and abnormal seedlings for germination were done according to the criteria accepted by International Seed Testing Association (ISTA) (2026). Seedlings were considered as normal if they possessed all essential structures; otherwise, they were classified as abnormal based on standard germination criteria.

#### Emergence tests

The sand used in germination tests was sieved through a one-millimeter mesh sieve, washed five times with tap water, and autoclaved at 121 °C for 60 min. Plastic germination test boxes were used. The sand was put into the test boxes and 100 treated and untreated seeds per each box were placed on the sand. Afterwards, they were covered with sterile sand and kept at 24 °C under 12 photoperiods. The experiments were conducted using a completely randomized design, with four replicates for each fungicide and dose. The boxes were watered at a few days intervals by considering the humidity. Ten days after the sowing, emerged plants from the treated/untreated seeds with the fungicides on the sand surface were considered as emerging plants. Fifteen days after the sowing, plant heights of the emerged seedlings were measured from the soil surface to the end. The number of emerged plant and their heights were calculated for each fungicide application and dose. The same calculations were also performed for the controls as well.

### Statistical analysis

Disease severity was determined according to Townsend & Heuberger (1943) and the percentage effect was determined according to Abbott (1925). Data were subjected to analysis of variance (ANOVA, San Francisco, CA, USA) according to a completely randomized design and means separated using the Tukey test. Statistical analysis was carried out using SPSS 16.0 (SPSS Inc., Chicago, IL, USA) software program. Percentage inhibition data were converted to proportions and transformed into probit units following Finney’s method. Probit regression analysis was applied by modeling probit-transformed inhibition values against log₁₀-transformed fungicide concentrations. Regression parameters were calculated using Microsoft Excel, and EC₅₀ values were derived as the concentrations corresponding to probit = 5. For fungicides in which inhibition levels did not reach 50% within the tested concentration range, EC₅₀ values were not determined.

## Results

### *In vitro* experiments

#### Effects of the treatments on conidium germination of *P. griseola*

The fungicides, biological preparation, and also their concentrations had significant (*P* ≤ 0.01) effects on conidium germination of *P. griseola* f. *griseola*. In addition, interactions between fungicides/biological preparation and concentrations were significant statistically. The *in vitro* results revealed a significant fungicide and biological preparation × concentration interaction, indicating that the inhibitory effects on conidial germination of *P. griseola* f. *griseola* varied depending on both the type of treatment and the applied concentration. At higher concentrations (100–10 µg mL^−1^), all fungicides exhibited strong inhibitory effects, with difenoconazole, azoxystrobin, and mancozeb providing the highest levels of inhibition (generally above 80–90%). However, as the concentration decreased, differences among fungicides became more pronounced. Difenoconazole and azoxystrobin maintained relatively high efficacy even at lower concentrations, whereas chlorothalonil and thiram showed a more substantial reduction in inhibitory activity. Mancozeb also exhibited a gradual decline in effectiveness with decreasing concentration, although it remained relatively effective across a broad range. In contrast, the *Trichoderma harzianum*-based treatment showed moderate inhibition (approximately 34–49%) across all concentrations, with less variation compared to chemical fungicides. These results demonstrate that the antifungal performance of the tested treatments is strongly concentration-dependent, and that comparisons among treatments should be made within the same concentration levels rather than based on overall averages (Table 2).

**Table 2:** Effects of the treatments on conidium germination of *P. griseola f. griseola in vitro* (mean ± standard errors).

Control	100	50	30	10	3	1	0.3	0.1	0.03	0.01
**Chlorothalonil**										
100 ± 0.0 ^Z^	13 ± 0.9^UY^	20 ± 1.3^PY^	25 ± 0.6^IT^	29 ± 1.3^GQ^	31 ± 2.0^FO^	33 ± 2.0^FK^	37 ± 1.1^DG^	43 ± 5.1^DE^	44 ± 2.8^DE^	56 ± 1.3^AB^
**% Effect**	87 ± 0.9	80 ± 1.3	76 ± 0.6	72 ± 1.3	70 ± 2.0	68 ± 2.0	63 ± 1.1	57 ± 2.8	57 ± 5.1	44 ± 1.3
**Mancozeb**										
100 ± 0.0 ^Z^	13 ± 1.0^VY^	14 ± 0.6^UY^	17 ± 0.9^RY^	18 ± 1.1^RY^	22 ± 0.6^MV^	30 ± 1.0^FP^	32 ± 0.9^FM^	32 ± 1.6^FL^	34 ± 1.3^EJ^	34 ± 2.3^EJ^
**% Effect**	88 ± 1.0	88 ± 1.0	83 ± 0.9	82 ± 1.1	79 ± 0.6	71 ± 1.0	68 ± 1.3	68 ± 1.6	66 ± 1.3	66 ± 2.3
**Difenoconazole**										
100 ± 0.0^Z^	10 ± 0.6^YZ^	11 ± 1.1^WZ^	14 ± 1.1^UY^	15 ± 1.7^TY^	20 ± 0.9^OX^	20 ± 3.3^OX^	24 ± 2.1^JT^	26 ± 1.1^HS^	29 ± 1.0^GQ^	32 ± 1.4^FM^
**% Effect**	91 ± 0.6	89 ± 1.1	86 ± 1.1	86 ± 1.7	80 ± 0.9	80 ± 3.3	76 ± 2.1	74 ± 1.1	72 ± 1.0	68 ± 1.4
**Thiram**										
100 ± 0.0^Z^	16 ± 1.8^SY^	17 ± 2.5^RY^	18 ± 1.1^RY^	23 ± 3.5^KU^	25 ± 0.9^HS^	27 ± 1.1^GR^	29 ± 0.6^GQ^	31 ± 0.8^FN^	35 ± 1.3^EI^	55 ± 2.1^BC^
**% Effect**	84 ± 1.8	83 ± 2.5	82 ± 1.1	77 ± 3.5	75 ± 0.9	73 ± 1.1	72 ± 0.6	69 ± 0.8	65 ± 1.3	45 ± 2.1
**Azoxystrobin**										
100 ± 0.0^Z^	11 ± 1.0^XZ^	12 ± 0.7^VY^	13 ± 0.9^UY^	18 ± 0.9^RY^	20 ± 1.2^PY^	21 ± 1.6^NW^	22 ± 1.1^LV^	29 ± 0.6^GQ^	31 ± 1.0^FO^	35 ± 1.3^EI^
**% Effect i**	90 ± 1.0	88 ± 0.7	87 ± 0.9	82 ± 0.9	81 ± 1.2	79 ± 1.6	78 ± 1.1	72 ± 0.6	70 ± 1.0	65 ± 1.3
**Carbendazim**										
100 ± 0.0^Z^	13 ± 2.0^UY^	16 ± 0.6^SY^	19 ± 1.0^QY^	20 ± 1.9^PX^	22 ± 1.3^KV^	27 ± 0.6^GR^	32 ± 0.9^FL^	34 ± 1.1^EJ^	36 ± 1.8^DH^	40 ± 0.9^DF^
**% Effect**	87 ± 2.0	85 ± 0.7	82 ± 1.0	80 ± 1.9	78 ± 1.3	73 ± 0.6	68 ± 0.9	66 ± 1.1	65 ± 1.8	60 ± 0.9
***Trichoderma harzianum***										
100 ± 0.0^Z^	46 ± 1.9^CD^	54 ± 5.0^BC^	56 ± 4.2^AB^	57 ± 2.5^AB^	58 ± 3.2^AB^	59 ± 2.5^AB^	61 ± 1.3^AB^	61 ± 1.7^AB^	62 ± 1.1^AB^	66 ± 2.4^A^
**% Effect**	49 ± 2.5	44 ± 4.2	43 ± 2.5	43 ± 3.6	42 ± 2.5	42 ± 2.5	40 ± 1.7	39 ± 1.3	38 ± 1.1	34 ± 2.4

**Note:**

Means followed by different letters indicate significant differences within each concentration (*P* ≤ 0.01).

**Figure 2:**
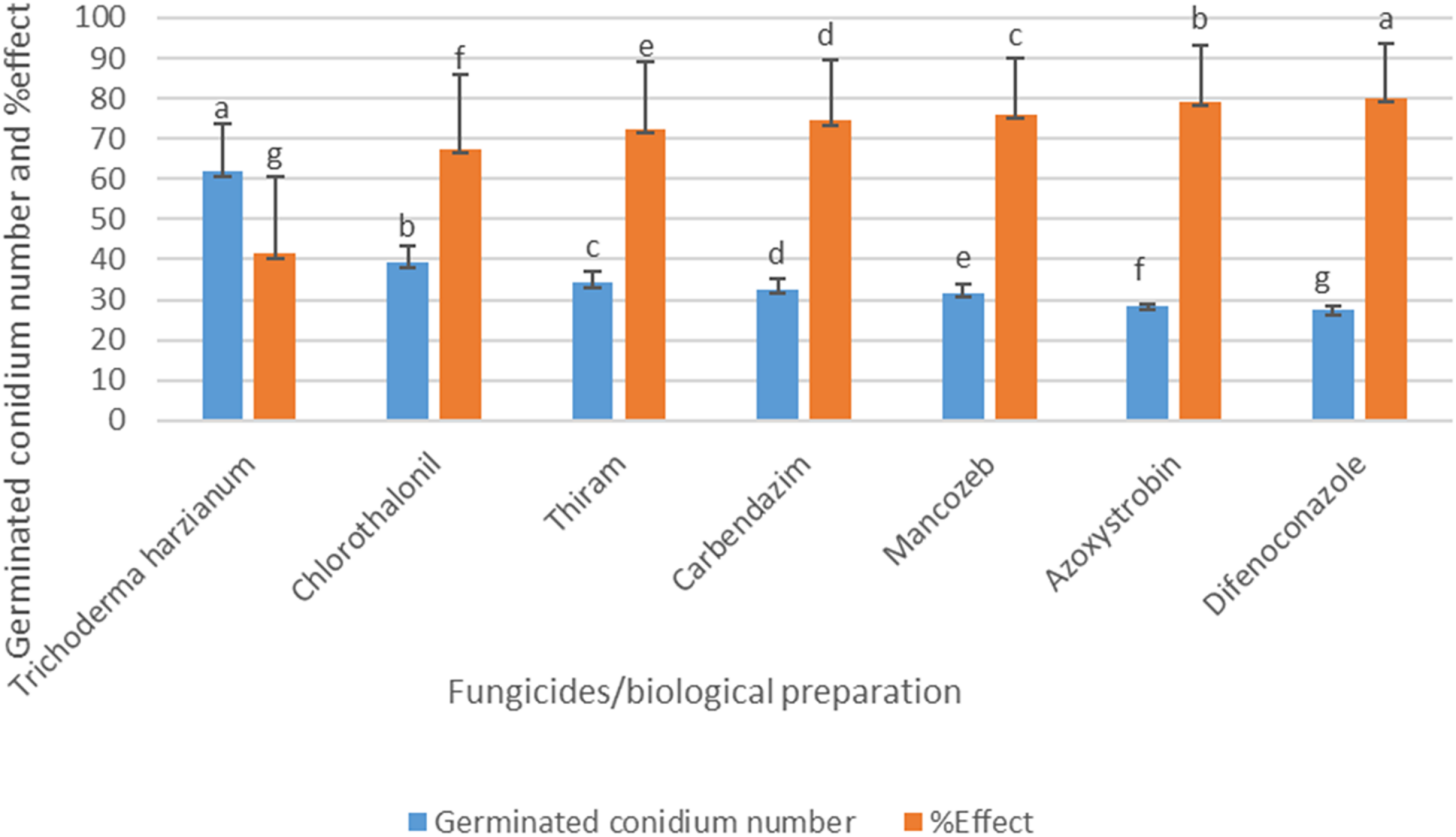
Comparison of efficacy of the treatments on conidium germination of *P. griseola f*. *griseola in vitro*. The same letters or the symbols are not significantly different at *P* ≤ 0.01. Error bars with standard errors.

To separately compare each application, the data at the Table 2 were further analysed as overall means of the each application. There were significant differences in germinated conidium numbers and percentage effects of the applications. For example, mean germinated conidium number was 32.6 at carbendazim application, whereas it was 61.7 at the biological preparation (*Trichoderma harzianum*) application. Conversely, percentage effects of carbendazim and the biological preparation were 74.4% and 41.4%, respectively. The highest mean percentage effect (80.2%) was found at difenoconazole application, followed by azoxystrobin, mancozeb, carbendazim, thiram, chlorothalonil and *Trichoderma harzianum*, respectively (Fig. 2).

**Figure 3:**
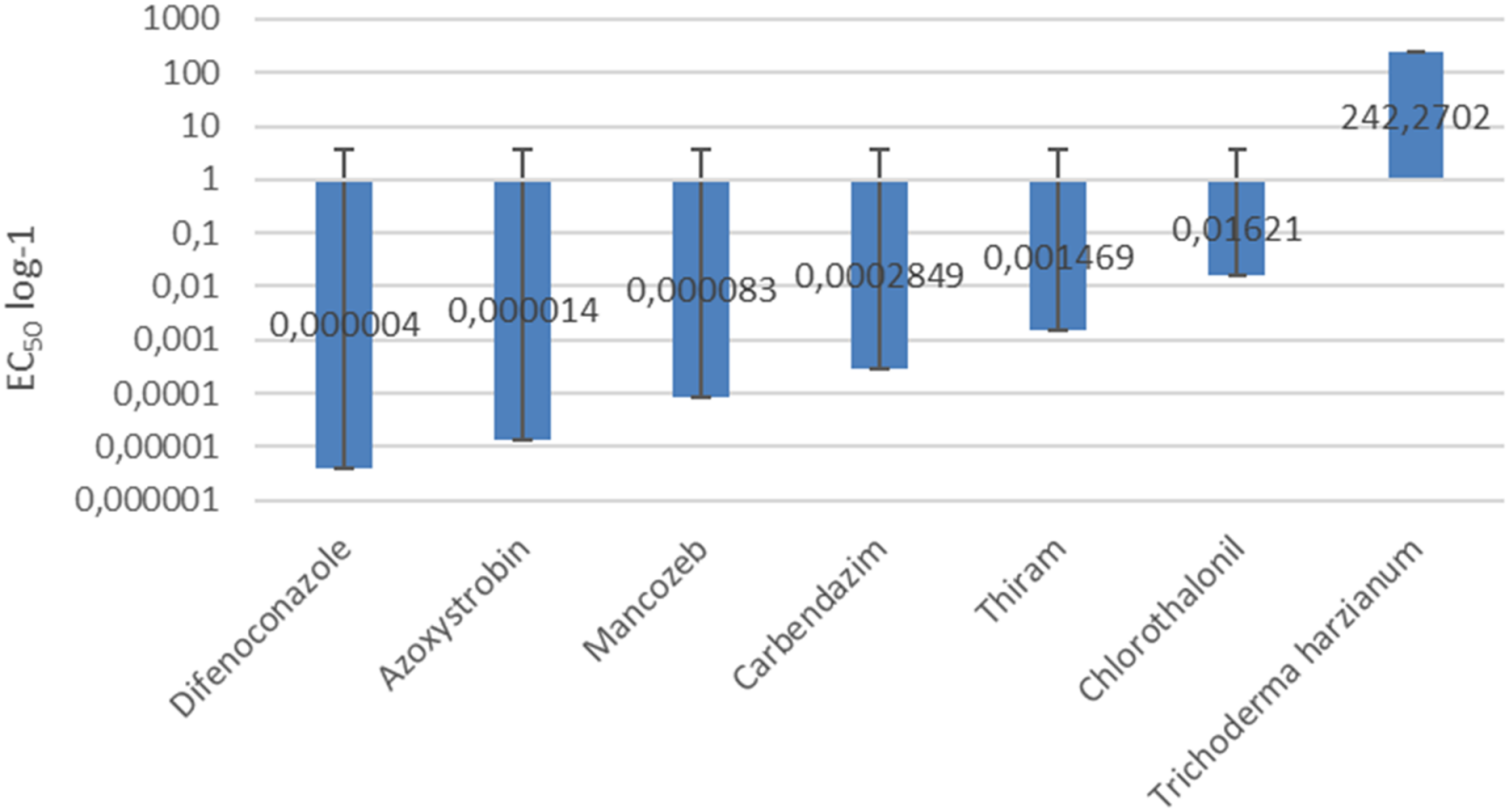
Susceptibility of conidial germination of *P. griseola f*. *griseola* to fungicides (EC_50_ values) (µg mL^−1^).

The ranking for the percentage effects of the fungicides were also confirmed by their EC_50_ values ranging from 0.000004 to 242.2702. Fungicide concentrations were expressed as µg of active ingredient per mL (Fig. 3).

In addition, to compare each dose of the applications, the data at the Table 2 were also further analysed as overall means of each concentration of the applications. Significant differences were detected in germinated conidium numbers between the concentrations of the applications. The highest germinated conidium number (100) was found at control, while the lowest one (10.06) was found at the 100 µg mL^−1^ concentrations of the applications.

### *In vivo* experiments

#### Effects of the treatments on seed germination of common bean

There were significant differences in germination rates among the applications. The highest germination rates, 98.5% and 98.7%, were at controls, but the lowest germination rate (75.2%) was found at the higher rate of carbendazim. The other lowest germination rate, 75.5%, were detected at the high dose of Chlorothalonil and the lower dose of carbendazim. However, the differences between fungicide applications were not significant statistically. Germination rates of the lower and higher doses of the biological preparation (*T. harzianum*) were 87.5% and 85.25%, respectively. All fungicides and the biological preparation significantly reduced germination compared to the control (*P* ≤ 0.05). Among the fungicides, the lowest reduction rate (20.5%) in germination was at the low dose of mancozeb application, but the high dose of carbendazim application caused the highest reduction rate (23.7%). When compared abnormal germination rates, the lowest abnormal germination rates (0.7% and 1.0%) were at controls. Compared to controls, there were significant differences in abnormal germination rates among the applications. difenoconazole application created the highest abnormal germination rates, 6.75 and 8% in cv. Gina. There were no significant differences between most of the applications, but compared to the controls, dramatic increases in abnormal germination of the seeds were founded. For example, increases in abnormal germination rates caused by difenoconazole applications were 575% and 966% at the low and high doses, respectively (Table 3).

**Table 3:** Seed germination rates and abnormal germination rates of common bean cv. Gina treated with fungicides and biological preparation (mean ± standard errors).

Fungicides	Germination rates (%)	Reduction in germination rate (%)	Abnormal germination rate (%)	Increase in abnormal germination rate (%)
Chlorothalonil (1^st^ dose)	77.0 ± 0.7 c	21.8	1.7 ± 0.4 bc	75
Chlorothalonil (2^nd^ dose)	75.5 ± 0.1 c	23.5	2.4 ± 0.5 bc	219
Difenoconazole (1^st^ dose)	77.7 ± 0.9 c	21.1	6.7 ± 1.4 a	575
Difenoconazole (2^nd^ dose)	77.2 ± 0.8 c	21.7	8.0 ± 1.9 a	966
Azoxystrobin (1^st^ dose)	77.2 ± 0.8 c	21.6	3.5 ± 0.9 b	250
Azoxystrobin (2^nd^ dose)	76.0 ± 0.3 c	23.0	3.2 ± 0.8 bc	333
Mancozeb (1^st^ dose)	78.2 ± 1.0 c	20.5	3.2 ± 0.8 bc	225
Mancozeb (2^nd^ dose)	78.0 ± 0.8 c	21.0	3.5 ± 0.8 b	366
Carbendazim (1^st^ dose)	75.5 ± 0.1 c	23.3	2.0 ± 0.4 bc	100
Carbendazim (2^nd^ dose)	75.2 ± 0.1 c	23.7	3.0 ± 0.7 bc	299
Thiram (1^st^ dose)	77.5 ± 0.8 c	21.3	2.5 ± 0.6 bc	150
Thiram (2^nd^ dose)	77.0 ± 0.6 c	22.0	3.0 ± 0.7 bc	299
*Trichoderma harzianum* (1^st^ dose)	87.5 ± 3.6 b	11.2	1.5 ± 0.3 bc	50
*Trichoderma harzianum* (2^nd^ dose)	85.2 ± 2.5 b	13.67	1.7 ± 0.4 bc	133
Control (1^st^ dose)	98.5 ± 6.3 a	–	1.0 ± 0.1 bc	–
Control (2^nd^ dose)	98.7 ± 7.7 a	–	0.7 ± 0.1 c	–

**Note:**

Values in each column are means of four replications and the same letters are not significant (*P* < 0.01).

#### Effects of the treatments on emergence and seedling growth of common bean

There were significant differences in emergence rates among the applications. The highest emergence rates (98.0% and 99.2%) were at controls, while the lower one (75.0%) was found at the high doses of mancozeb and carbendazim. Emergence rates were 87.2% and 86.2% at the lower and higher doses of the biological preparation (*Trichoderma harzianum*). Compared to controls, all fungicide applications including the biological preparation led to significant reductions in emergence rates of cv. Gina. The higher doses of carbendazim and mancozeb resulted in the highest reduction rate (24.4%) in emergence of cv. Gina, but the lower ones, 10.9% and 13.0%, were at the lower and higher doses of the biological preparation applications. With regard to seedling heights, the highest seedling heights (20.2 and 23.0 mm) were at controls. There were significant differences in seedling heights among the applications. For example, seedling heights of the seeds were 14.8 and 10.5 mm at the lower doses of chlorothalonil and Thiram, respectively, whereas they were 11 and 9.7 mm at the higher doses of those fungicides, respectively. Compared to controls, all fungicide and the biological preparation applications significantly reduced seedling heights of cv. Gina. The reductions in seedling height ranged from 11.1% to 68.4% among the applications (Table 4).

**Table 4:** Emergence rates and seedling heights of common bean cv. Gina treated with fungicides and biological preparation (mean ± standard errors).

Fungicides	Emergence rates (%)	Reduction in emergence rate (%)	Plant heights (mm)	Reduction in plant height (%)
Chlorothalonil (1^st^ dose)	81.5 ± 2.1 cde	16.8	14.8 ± 1.6 b–e	26.9
Chlorothalonil (2^nd^ dose)	80.6 ± 1.8 de	18.7	11.0 ± 1.6 def	52.1
Difenoconazole (1^st^ dose)	78.2 ± 1.2 ef	20.1	11.5 ± 1.6 def	43.2
Difenoconazole (2^nd^ dose)	77.0 ± 1.4 ef	22.4	7.2 ± 1.7 f	68.4
Azoxystrobin (1^st^ dose)	86.2 ± 3.7 bc	11.9	15.2 ± 2.4 b–e	24.6
Azoxystrobin (2^nd^ dose)	82.2 ± 2.3 b–e	17.1	14.2 ± 2.3 cde	38.0
Mancozeb (1^st^ dose)	77.2 ± 1.2 ef	21.1	18.0 ± 2.8 abc	11.1
Mancozeb (2^nd^ dose)	75.0 ± 1.3 f	24.4	14.0 ± 2.2 cde	39.1
Carbendazim (1^st^ dose)	88.3 ± 3.0 bcd	9.8	14.2 ± 2.4 cde	29.6
Carbendazim (2^nd^ dose)	75.0 ± 1.3 f	24.4	14.3 ± 2.4 b–e	37.6
Thiram (1^st^ dose)	79.7 ± 1.5 def	18.6	10.5 ± 1.7 ef	48.1
Thiram (2^nd^ dose)	78.0 ± 1.2 ef	21.4	9.7 ± 1.7 ef	57.6
*Trichoderma harzianum* (1^st^ dose)	87.2 ± 4.0 b	10.9	16.7 ± 2.6 bcd	17.2
*Trichoderma harzianum* (2^nd^ dose)	86.2 ± 3.5 bc	13.0	13.2 ± 1.9 cde	
Control (1^st^ dose)	98.0 ± 5.9 a	–	20.2 ± 3.3 ab	–
Control (2^nd^ dose)	99.2 ± 7.2 a	–	23.0 ± 3.9 a	–

**Note:**

Values in each column are means of four replications and the same letters are not significant (*P* < 0.01).

**Figure 4:**
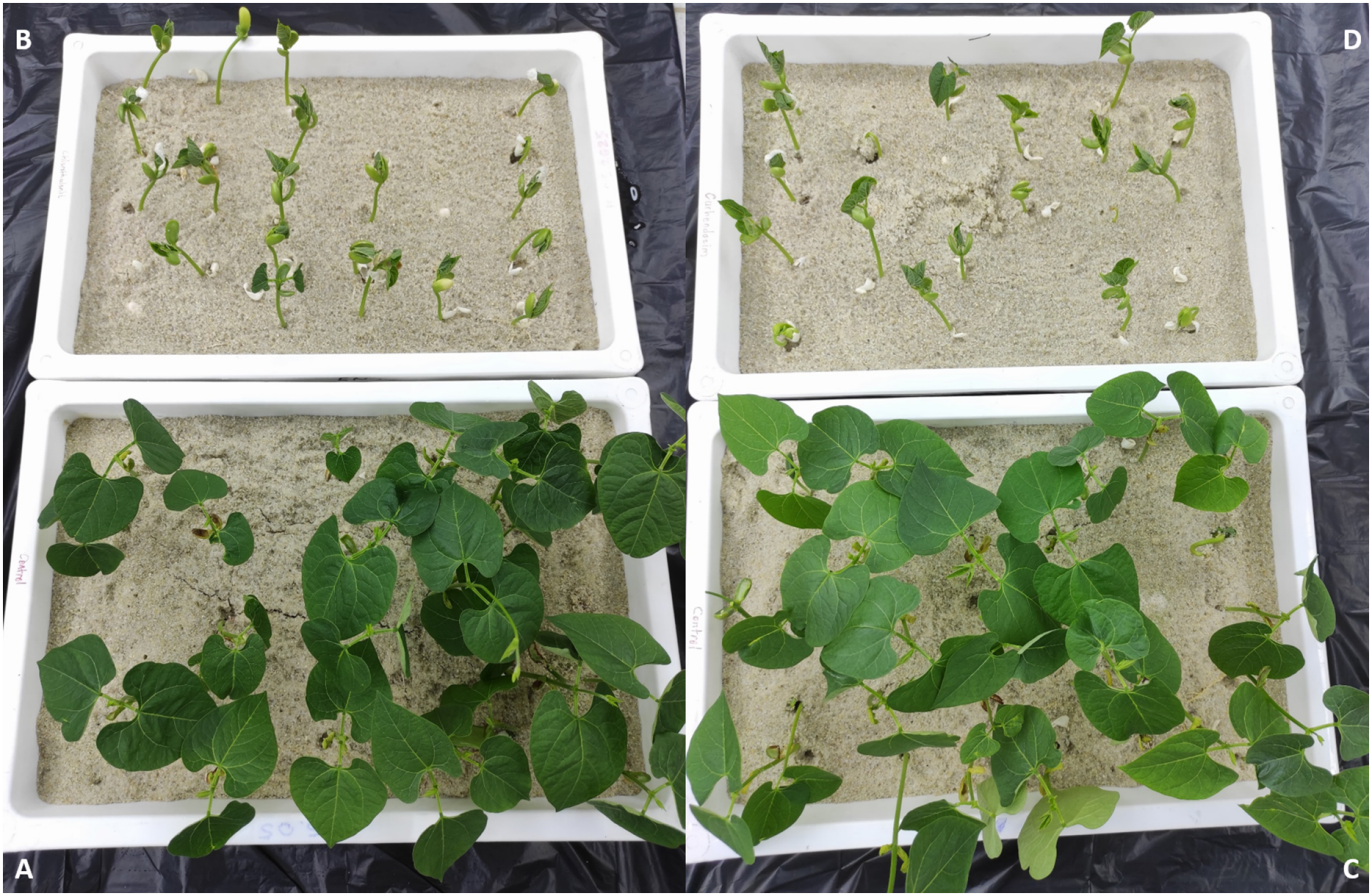
Emergence and seedling growth of common bean at control (A, C) and emergence and seedling growth of common bean with the lower dose (0.4 g/kg seed) of Chlorothalonil (B), (0.08 g/kg seed) of Carbendazim (D).

**Figure 5:**
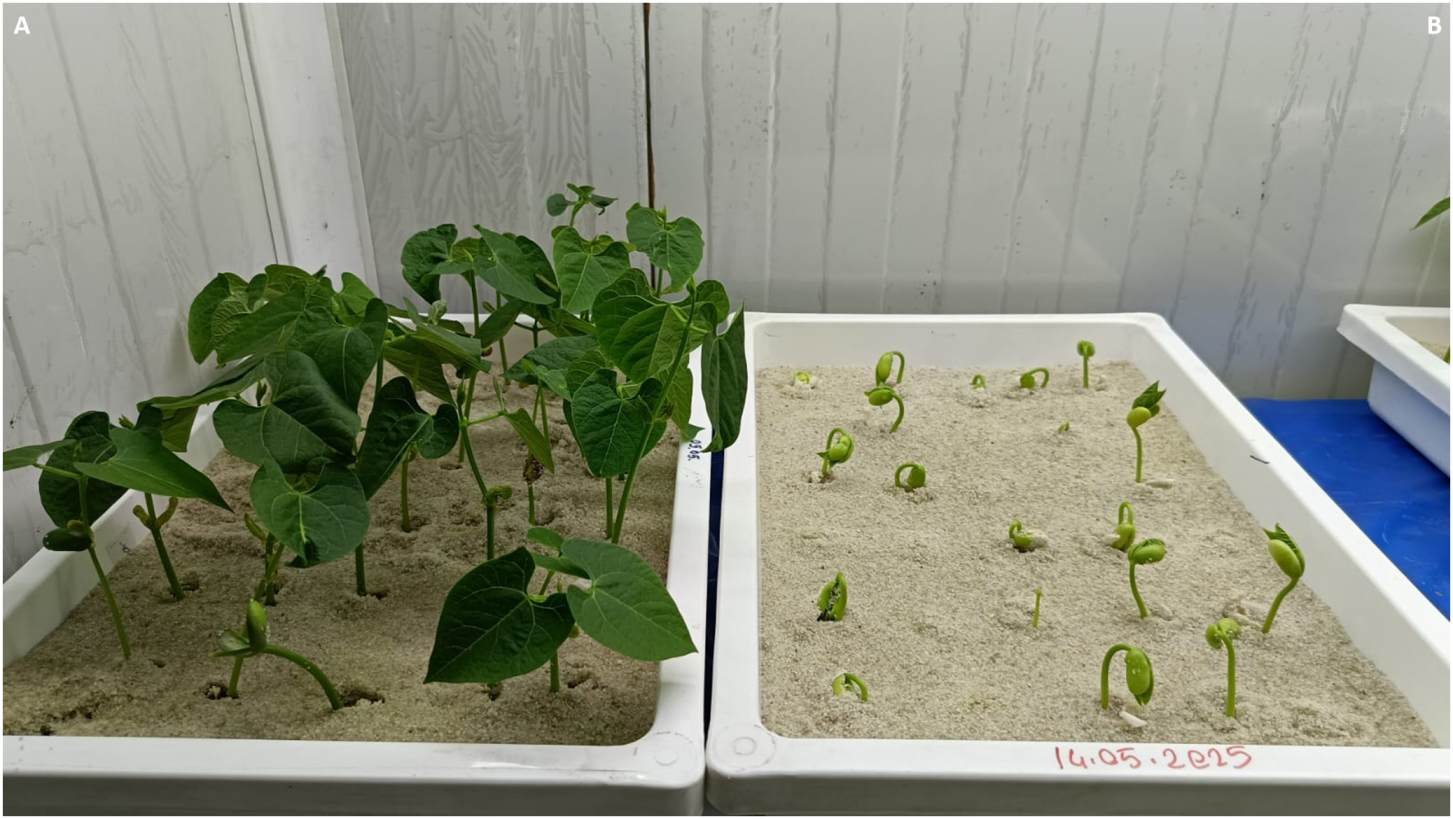
Emergence and seedling growth of common bean at control (A). Emergence and seedling growth of common bean with the lower dose (0.16 g/kg seed) of Difenoconazole (B).

In addition, three examples related to emergence and seedling growth of common bean were given for *in vivo* experiments in Figs. 4 and 5.

Compared to the control, seed treatment with the lower dose of chlorothalonil caused reductions, 16.8% and 26.9%, carbendazim 9.8% and 29.6%, in emergence and seedling heigth of common bean, respectively.

Compared to the control, seed treatment with the lower dose of difenoconazole caused reductions, 20.1% and 43.2%, in emergence and seedling height of common bean.

## Discussion

*P. griseola*, the causal agent of angular leaf spot disease of common bean, is a seedborne fungus and seed treatments with fungicides is the main control practice that can be used for the management of the disease (Ayesha et al., 2021; PlantwisePlus, 2025). However, several researchers (Fontem, Nziengui-Nziengui & Bikoro, 2007; Mongi et al., 2018) also reported that the disease could be prevented with foliar spraying of certain fungicides such as mancozeb, carbendazim, chlorothalonil, difeconazole and thiophanate-methyl in the field. However, considering the seedborne nature of *P. griseola*, seed treatments with fungicides are crucial for the control of the fungus (Ayesha et al., 2021). Various fungicides (*e.g*., carbendazim, azoxystrobin, difenoconazole, propiconazole, tebuconazole, flutriafol mancozeb, metalaxyl, mancozeb, chlorothalonil, tridemorph, dinocap, fentin hydroxide thiophanate methyl) have been used to control *P. griseola* (Rajappan & Yesuraja, 1999; Pamela, Mawejje & Ugen, 2014; Hennipman et al., 2019; Chandrashekara et al., 2022; Wani et al., 2022; Gorshkov et al., 2023), but in the present study, six commercial fungicides with different action mechanism and chemical groups were selected to examine their effects on both *P. griseola* in the *in vitro* and common bean in the *in vivo* based on seed treatments.

The *in vitro* results of the *in vitro* experiments of the present study showed that all the tested commercial fungicides (difenoconazole, azoxystrobin, mancozeb, carbendazim, thiram, and chlorothalonil) significantly reduced the germination of *P. griseola* f. *griseola*, indicating their inhibitory antifungal strength. However, percentage effects of the tested fungicides on the conidium germination were ranked from the highest to the lowest one as follows: difenoconazole, azoxystrobin, mancozeb, carbendazim, thiram, and chlorothalonil, respectively. In a previous study (Sartorato, 2007), of fungicides (benomyl, fluazinam, chlorothalonil and mancozeb) investigated, fluazinam and chlorothalonil had the lowest inhibition strength on conidium germination and mycelium growth of *P. griseola*, supporting the results of the present study. In another study, Bárcena et al. (2015) found that Tricyclazole inhibited growth of *P. griseola* by reducing melanin synthesis of the fungus *in vitro*. It was also previously reported that azoxystrobin prevented conidium germination and mycelium growth of various fungi (Zhang et al., 2010). In another *in vitro* study, some fungicides (*e.g*., fenamidone, mancozeb, pyraclostrobin, metiram, azoxystrobin, difenoconazole, trifloxystrobin, tebuconazole) inhibited growth of *P. griseola* (Kansal Sandeep, 2016). Apart from the fungicides, metabolites excreted by *Trichoderma* species (*T*. *harzianum, T. asperellum, T. endophyticum, T. lentiforme, T. koningiopsis* and *T. viride*) could significantly reduce mycelial growth and conidium numbers of *P. griseola* in the *in vitro*, indicating their antagonistic effects against *P. griseola* as well (Scortegagna et al., 2024). However, antifungal effects of the *Trichoderma* species could be lower than fungicides, likewise, results of the present study showed that compared to the tested six fungicides, biological preparation (*T. harzianum*) had the lowest percentage inhibitory effect against *P. griseola* f. *griseola*.

Although the results of the *in vitro* experiments of the present study revealed inhibition strengths of the tested six fungicides against conidium germination of *P. griseola*, they also resulted in significant reductions ranging from 20.5% to 23.7% in germination rates of common bean seeds compared to controls *in vivo* experiments. In this regard, Fatma, Kamal & Srivastava (2017) reported that compared to control, seeds treatments with Mancozeb led to decreases at the rates of 5% and 11.67% in germination of black gram (*Vigna mungo* L.) and green gram (*Vigna radiata* L.), respectively. Tort & Türkyılmaz (2003) reported that seeds treatments with Captan led to 13.33% and 26.66% reductions in germination of pepper, similarly Murthy & Sudarshana (2003) stated that Metalaxyl applications created reductions up to 42.5% in germination of *Cajanus cajan*. These results are in agreement with the results of the present study. With regard to abnormal germination, compared to controls, difenoconazole application caused the highest abnormal germination rates, 6.75% and 8% in common bean (cv. Gina). The findings of the present study and the others mentioned before indicate phtotoxicity of the fungicides to those tested plants.

Compared to the control group, seed treatments with the six commercial fungicides (difenoconazole, azoxystrobin, mancozeb, carbendazim, thiram, and chlorothalonil) significantly reduced the emergence rates of common bean (cv. Gina) by up to 24.4%. However, the biological preparation (*T. harzianum*) resulted in the lowest reductions. In addition to negative effects on emergence of common bean, compared to controls, the tested fungicides also caused to significant reductions ranging from 11.1% to 68.4% in seedling heights, revealing their phytotoxicity to common bean. With respect to legume, tebuconazole led to reductions in shoot growth, chlorophyll and protein content of chickpea to plants (Ahemad, 2011), while seed treatments with fungicides (carbendazim + thiram) reduced seedling length up to 19.73% in soybean (Carvalho et al., 2020). As regards other legumes, Fatma, Kamal & Srivastava (2017) and Fatma et al. (2018) underscored that increasing doses of mancozeb led to higher phytotoxicity to legumes such as black gram (*Vigna mungo* L.) and green gram (*Vigna radiata* L.). Similarly, dose increases of tetramethylthiuram also resulted in structural alterations in cell walls and phototoxicity to pea (*Pisum sativum* L.) (Gorshkov et al., 2020). Moreover, applications of carbendazim, kitazin and hexaconazole led to alterations in anatomy and physiology and induced cytotoxicity and cellular damage to pea (Shahid et al., 2018), several fungicides (*e.g*., difenoconazole, propiconazole, tebuconazole, flutriafol) adversely affected cell walls of pea plants by altering synthesis of cellulose microfibrils and amount of matrix polysaccharides (Gorshkov et al., 2023). Recommended application dose of tebuconazole caused phytotoxicity to several legumes such as chickpea, pea, lentil and greengram (Ahemad, 2011). In *Phaseolus vulgaris* plants, even below the recommended doses of azoxystrobin and carbendazim crop injury and physiological disorders by negatively affecting chlorophyll content and photosynthesis (Grecco et al., 2024). Similarly, in the present study, lower doses of the tested six commercial fungicides (difenoconazole, azoxystrobin, mancozeb, carbendazim, thiram, and chlorothalonil) than their recommended rates also led to distinctive phytotoxicity to common bean (cv. Gina).

Fungicide-based seed treatments may exert unintended effects on non-target organisms, particularly seed-associated endophytes, which play an important role in seed vigor and early plant development. Disruption of these beneficial microbial communities may partly explain the reductions observed in germination and seedling growth, indicating that fungicide effects are not solely due to direct phytotoxicity but may also involve indirect microbiome-mediated mechanisms (Ayesha et al., 2021). This highlights the need to consider the seed microbiome as an integral component of plant health when evaluating fungicide applications.

In light of these limitations, increasing attention has been directed toward alternative approaches that combine efficacy with environmental safety. Natural products, physical treatments, and biological control agents have emerged as promising options, as they tend to exert the lower toxicity on both plants and non-target organisms (Moumni, Brodal & Romanazzi, 2023). In particular, so-called “basic substances” approved under Regulation (EC) No 1107/2009, such as chitosan, vinegar, and hydrogen peroxide, have shown antifungal activity against seed-borne pathogens and represent a regulatory-supported pathway toward reduced chemical dependency. However, their efficacy under field conditions, persistence, and consistency across different pathosystems remain key challenges that require further optimization (Orzali et al., 2023).

From a broader disease management perspective, reliance solely on chemical seed treatments appears increasingly unsustainable. Instead, integrating alternative treatments with host resistance and cultural practices offers a more resilient and environmentally sound strategy. The use of resistant cultivars, combined with optimized agronomic practices, can reduce disease pressure and minimize the need for chemical inputs (Taboada et al., 2022). Therefore, future research should focus on developing integrated seed treatment strategies that preserve beneficial microbiota, maintain plant health, and ensure effective control of seed-borne pathogens.

## Conclusions

The present study for the first time examined effects of six commercial fungicides (difenoconazole, azoxystrobin, mancozeb, carbendazim, thiram, and chlorothalonil) with different action mechanism and chemical groups on both *P. griseola* in the *in vitro* and common bean in the *in vivo* based on seed treatments. In conclusion, this study demonstrated that the tested fungicides exhibited strong inhibitory effects on the conidial germination of *P. griseola* under *in vitro* conditions; however, their *in vivo* application as seed treatments resulted in varying degrees of phytotoxicity on common bean. These findings highlight a critical trade-off between antifungal efficacy and crop safety, emphasizing that effective disease control does not necessarily guarantee optimal plant performance.

From a broader perspective, the observed responses may also be influenced by genotypic variation among common bean cultivars. Since only a single cultivar (cv. Gina) was evaluated in this study, it is likely that different genotypes may exhibit varying levels of tolerance or sensitivity to fungicide treatments. Therefore, future studies incorporating multiple cultivars are essential to better understand host-dependent responses and to develop more targeted and cultivar-specific disease management strategies.

The results of this study also have important practical implications for disease management. While chemical fungicides remain effective tools for controlling *P. griseola*, their use, particularly as seed treatments, should be carefully optimized in terms of dose, formulation, and application method to minimize phytotoxic effects. In this context, the moderate but stable performance of the *Trichoderma harzianum*-based treatment, combined with its lack of phytotoxicity, suggests that biological control agents may serve as safer alternatives or complementary components within integrated disease management programs.

Overall, these findings underscore the importance of adopting an integrated approach that combines chemical and biological control strategies, along with consideration of host genotype, to achieve sustainable and effective management of angular leaf spot. Further field-based studies are required to validate these results under diverse environmental conditions and to translate them into practical agricultural recommendations.

## Supplemental Information

10.7717/peerj.21477/supp-1 Supplemental Information 1 Raw data for all the tables given in the text. Each data point shows statistical values for conidia germination numbers, seed germination rates, abnormal germination rates, seed emergence rates, and plant heights. All the fungicides significantly inhibited conidium germination *in vitro* and their effectiveness were ranked from the highest to the lowest on tables. Fungicide treatments also significantly increased abnormal germination rate.

## Additional Information and Declarations

### Data Availability

The following information was supplied regarding data availability:

The raw data is available in Supplemental File.
